# Inactivation of SARS CoV-2 on porous and nonporous surfaces by compact portable plasma reactor

**DOI:** 10.3389/fbioe.2024.1325336

**Published:** 2024-02-29

**Authors:** Bhaswati Choudhury, John A. Lednicky, Julia C. Loeb, Sherlie Portugal, Subrata Roy

**Affiliations:** ^1^ SurfPlasma, Inc., Gainesville, FL, United States; ^2^ Emerging Pathogens Institute, University of Florida, Gainesville, FL, United States; ^3^ Department of Environmental and Global Health, University of Florida, Gainesville, FL, United States; ^4^ School of Electrical Engineering, Technological University of Panama, Panama City, Panama; ^5^ Department of Mechanical and Aerospace Engineering, University of Florida, Gainesville, FL, United States

**Keywords:** plasma, COVID-19, SARS CoV-2, ozone, disinfection, sterilization, dielectric barrier discharge

## Abstract

We report the inactivation of SARS CoV-2 and its surrogate—Human coronavirus OC43 (HCoV-OC43), on representative porous (KN95 mask material) and nonporous materials (aluminum and polycarbonate) using a Compact Portable Plasma Reactor (CPPR). The CPPR is a compact (48 cm^3^), lightweight, portable and scalable device that forms Dielectric Barrier Discharge which generates ozone using surrounding atmosphere as input gas, eliminating the need of source gas tanks. Iterative CPPR exposure time experiments were performed on inoculated material samples in 3 operating volumes. Minimum CPPR exposure times of 5–15 min resulted in 4–5 log reduction of SARS CoV-2 and its surrogate on representative material samples. Ozone concentration and CPPR energy requirements for virus inactivation are documented. Difference in disinfection requirements in porous and non-porous material samples is discussed along with initial scaling studies using the CPPR in 3 operating volumes. The results of this feasibility study, along with existing literature on ozone and CPPR decontamination, show the potential of the CPPR as a powerful technology to reduce fomite transmission of enveloped respiratory virus-induced infectious diseases such as COVID-19. The CPPR can overcome limitations of high temperatures, long exposure times, bulky equipment, and toxic residuals related to conventional decontamination technologies.

## 1 Introduction

The COVID-19 pandemic revealed the global need of potent, portable, and economical technologies for the effective decontamination of personal protective equipment (PPE), medical devices, and personal effects to reduce the spread of infectious diseases (Westover et al., 2022). Although the worldwide shortage of PPE that occurred during the early stages of the COVID-19 (coronavirus disease 2019) pandemic has been resolved, effective decontamination of PPE and medical devices by portable and economical technologies is still important for countries with limited resources, in emergency situations, in austere military medical environments, and for future outbreaks. The rise of hospital acquired infections (HAIs) causing around 75,000 deaths annually in the US alone (epi.dph.ncdhhs; Caselli et al., 2018), substantially adds to this need for potent decontamination technologies that can prevent the spread of infectious agents through contact with contaminated surfaces. Although conventional decontamination technologies like steam sterilization, ethylene oxide and hydrogen peroxide are effective, they are limited by high processing temperatures and pressures, long exposure times, material incompatibility, high cost, bulky equipment, and toxic residuals (Scholtz et al., 2015; Mandal et al., 2018; Choudhury et al., 2022; Epelle et al., 2023a). Alternate decontamination technologies are essential to overcome these limitations, especially with the increase in advanced heat-sensitive electronics and the rise in antibiotic resistant micro-organisms (Aslam et al., 2018; Choudhury et al., 2022). This paper presents a proof-of-concept study of the Compact Portable Plasma Reactor (CPPR) (Roy and Atencio, 2020) for inactivating Severe acute-respiratory coronavirus 2 (SARS-COV-2), the causative agent of the COVID-19 pandemic (Gorbalenya et al., 2020), and one of its commonly studied surrogates—Human coronavirus organ culture 43 (HCoV-OC43), on porous and nonporous materials in ambient conditions under varying operating parameters. The CPPR is based on cold plasma generated ozone, an alternative decontamination technology, that works under ambient conditions. Previous studies on CPPR have shown its effectiveness against various types of bacteria (Choudhury et al., 2018; Roy et al., 2021; Choudhury et al., 2023). The SARS-COV-2 inactivation results presented in this paper, along with previously published literature on CPPR and ozone (Kogelschatz, 2003; Park et al., 2006; Choudhury et al., 2018; Roy et al., 2021; Choudhury et al., 2023), establishes the potential of CPPR as a decontamination technology for preventing HAIs (hospital acquired infections), further spread of COVID-19 and possible future outbreaks.

The CPPR is a compact (48 cubic centimeters), lightweight (55 g), energy-efficient, and scalable device for surface Dielectric Barrier Discharge (DBD)-based *in-situ* ozone generation from atmospheric air with an ozone yield of 68.6 g/KWh (Choudhury et al., 2018). CPPR differentiates itself from commonly available DBD reactor systems which are associated with disadvantages of bulky equipment, low ozone production and high electrical power consumption (Kogelschatz, 2003; Park et al., 2006; Epelle et al., 2023a). Previous studies have established the effectiveness of CPPR against bacterial and fungal species including *Escherichia coli*, *Bacillus subtilis*, *Pseudomonas aeruginosa*, *Enterobacter* sp., *Chrysobacter* sp., *Aspergillus* sp., *Xanthomonas* sp., *Chromobacter* sp., *Fusarium* sp. and *Enterobacter* sp., and *Staphylococcus aureus* (Choudhury et al., 2018; Roy et al., 2021; Choudhury et al., 2023).

Plasma is a mixture of charged and neutral particles at equilibrium with zero net electrical charge. It is known as the fourth state of matter in physical sciences and is essentially an assembly of ions, electrons, neutral and excited atoms, molecules, radicals, and UV photons (Langmuir, 1928; Fridman et al., 2008). Cold plasma, also known as non-thermal plasma or atmospheric plasma, is a type of plasma created at or near room temperature, without heating the surrounding gas to high temperatures. Literature shows cold plasma to be a potential alternative to traditional decontamination methods used for food preservation, surface disinfection, medical device sterilization, and surface sterilization in space missions (Laroussi and Leipold, 2004; Ehlbeck et al., 2010; Weltmann et al., 2012; Mastanaiah et al., 2013; Rutala and Weber, 2013; Scholtz et al., 2015; Choudhury et al., 2018; Mandal et al., 2018; Gradini et al., 2019; Office of Planetary Protection, 2019; Bayarri et al., 2021; Feizollahi et al., 2021; Roy et al., 2021; Bhartiya et al., 2022; Choudhury et al., 2022; Ashokkumar et al., 2023; Epelle et al., 2023b; Choudhury et al., 2023; Kaushik et al., 2023). Dielectric Barrier Discharge (DBD) is a type of cold plasma which is formed when a high enough alternating voltage is applied across one or more electrodes separated by a dielectric medium. DBD formed in atmospheric air results in the generation of reactive oxygen and nitrogen species (RONS) like ozone due to ionization of the surrounding air (Kogelschatz, 2003; Bhartiya et al., 2022). Additionally, DBD generation can also be designed to create a localized electric field that can interact with the surrounding gas and modify its flow (Choudhury et al., 2022). DBD is classified as either volume DBD (VDBD) or surface DBD (SDBD) depending on where the DBD is formed. DBD decontamination occurs through direct contact with discharge or indirect contact with reactive species formed with the discharge (Choudhury et al., 2022). Indirect DBD treatment can treat hidden surfaces and surfaces larger than the discharge surface area. Literature shows that DBD decontamination can overcome limitations of high processing temperatures, long exposure times, material incompatibility, toxic residuals and low effectiveness associated with conventional disinfection technologies (Ehlbeck et al., 2010; Weltmann et al., 2012; Choudhury et al., 2018; Gradini et al., 2019; Office of Planetary Protection, 2019; Feizollahi et al., 2021; Roy et al., 2021; Choudhury et al., 2023). For example, steam sterilization is not suitable for heat sensitive materials, ethylene oxide requires long exposure times and is carcinogenic in nature, hydrogen peroxide decontamination involves high humidity levels that can cause moisture damage and UV treatment cannot be used to disinfect hidden surfaces (Rutala and Weber, 2013; Office of Planetary Protection, 2019; Epelle et al., 2023a; Epelle et al., 2023b; Choudhury et al., 2023). The CPPR uses atmospheric SDBD to generate reactive species like ozone which contributes to achieving microbial decontamination. Microbial decontamination by cold plasmas can be attributed to three components UV radiation, plasma temperature and reactive chemical species (Laroussi and Leipold, 2004). Literature shows that reactive chemical species is the main contributor in atmospheric SDBD microbial decontamination, while temperature and UV radiation do not play a major role (Laroussi and Leipold, 2004; Mastanaiah et al., 2013). Among other reactive species produced by SDBD at voltage and frequencies used by the CPPR, ozone has been found to be a primary contributor in microbial decontamination (Mastanaiah et al., 2013; Choudhury et al., 2023).

Ozone, one of the DBD generated RONS, is a strong antimicrobial agent with high oxidation potential (2.07 V) which makes it more effective in eradicating pathogens than many other chemicals on surfaces (Epelle et al., 2023b)–see Table 1. Ozone is known to be effective against a wide range of pathogens including bacteria, fungi, and viruses (Choudhury et al., 2018; Roy et al., 2021; Choudhury et al., 2022; Epelle et al., 2023a; Epelle et al., 2023b; Choudhury et al., 2023). Although there is a possibility of other RONS species contributing to CPPR decontamination, this study mainly focuses on ozone as the decontaminating chemical species due to its long lifespan compared to other RONS (Laroussi and Leipold, 2004; Mastanaiah et al., 2013) and its role as the primary contributor in decontamination for SDBD generated by reactors operating at voltage and frequencies similar to the CPPR (Mastanaiah et al., 2013; Choudhury et al., 2022; Choudhury et al., 2023).

**TABLE 1:** Oxidation potential of Ozone compared to other chemicals (Epelle et al., 2023b).

Oxidizing agent	Oxidation potential (eV)
Fluorine	3.06
Ozone	2.07
Permanganate	1.67
Chlorine dioxide	1.50
Hypochlorous acid	1.49
Chlorine gas	1.36

Ozone achieves inactivation of a target organism by a series of oxidation-reactions between ozone and biomolecules that make up the external and internal structures of a target organism (Bayarri et al., 2021). It inactivates enveloped viruses, like SARS-CoV-2, by altering the lipids and proteins present in the virus membranes, making them dysfunctional. Although literature on ozone inactivation of non-enveloped viruses is still unclear, researchers suggest that it is caused by damage in the capsid and/or genome of the virus. Excess or residual ozone post inactivation rapidly decomposes to oxygen due to the unstable nature of its molecular structure. This instability requires on-site ozone generation from atmospheric air eliminating the need of bulky gas tanks. Additionally, ozone has the penetration capability to reach hidden areas, obstructed surfaces and layered fabric (Epelle et al., 2023a; Choudhury et al., 2023). Although high ozone concentrations can oxidize rare-earth metals in sensitive electronics, ozone decontamination treatment of such materials can be performed with low ozone concentrations and high exposure times to minimize material damage during decontamination (Bayarri et al., 2021). Thus, ozone can be considered as a strong antimicrobial agent with excellent penetration capabilities and negligible toxic residuals.

Severe acute-respiratory coronavirus 2 (SARS-CoV-2), genus *Betacoronavirus*, subgenus *Sarbecovirus*, is a virus of the species Severe acute-respiratory syndrome–related coronavirus (Gorbalenya et al., 2020) and the causative agent of the COVID-19 (coronavirus disease 2019) pandemic. Human coronavirus OC43 (HCoV-OC43), genus *Betacoronavirus*, subgenus *Embecovirus*, is a SARS-CoV-2 surrogate. It is one of the viruses responsible for the common cold (Vabret et al., 2005). HCoV-OC43 is a member of the same genus as SARS-CoV-2, its replication and transmission processes are matched to SARS-CoV-2, and it is more resistant than other human coronaviruses to common disinfectants (Schirtzinger et al., 2022). HCoV-OC43 was identified by the American Society for Testing and Materials (ASTM) as a preferred SARS-CoV-2 surrogate to perform biosafety level (BSL)—2 research that can give insights on SARS-CoV-2 decontamination while avoiding the resource costs and safety concerns associated with SARS-CoV-2 experiments which require BSL-3 laboratory (Schirtzinger et al., 2022). The resources used for SARS-CoV-2 experiments in BSL-3 laboratory performed in this study was minimized by designing these experiments based on HCoV-OC43 inactivation data which was collected first.

This paper demonstrates a proof-of-concept study performed under the NSF-SBIR phase 1 program to establish the feasibility of CPPR technology to reduce the spread of infectious diseases like COVID-19 and future pandemics. Both SARS CoV-2 and and HCoV-OC43, were tested in this study. Iterative experiments on the HCoV-OC43 were first performed for selected porous and non-porous materials in 3 operating volumes to find the minimum exposure times required for inactivation by the CPPR in the 3 volumes. These exposure times were used to determine the initial exposure times for inactivation experiments on SARS CoV-2, followed by increments in exposure times till complete inactivation was achieved. This iterative approach of first testing the surrogate virus to determine the initial exposure times for inactivation experiments on SARS CoV-2 was important to reduce time in the BLS3 lab and save resources available for the project. Results show the inactivation of SARS CoV-2 and HCoV-OC43 on representative porous (KN95 mask material) and nonporous materials (aluminum metal and polycarbonate plastic) with the CPPR within a minimum exposure time of 5–15 min. Aditionally, a scaling study of HCoV-OC43 inactivation in three operating volumes—0.1 cu. ft (0.0028 cu. m), 0.2 cu. ft (0.0057 cu. m) and 0.3 cu. ft (0.0085 cu. m), using 1 CPPR, is reported. Ozone requirements in the operating volumes for the time of decontamination and CPPR energy requirements are also reported. This proof-of-concept study of SARS-COV-2 inactivation with the CPPR technology, literature on CPPR inactivation of various bacteria and the advantages associated with DBD based ozone decontamination, establishes the CPPR as a powerful decontamination technology to prevent the spread of infection diseases like COVID-19 and future pandemics. Further the scaling study reported here suggests that the CPPR decontamination technology can be scaled to meet a range of sterilization and disinfection needs. Potential applications include sterilization of PPE, medical devices, etc. in healthcare facilities, medical device companies, aerospace industry and food and beverage companies.

## 2 Materials and methods

### 2.1 Compact portable plasma reactor (CPPR)

**FIGURE 1:**
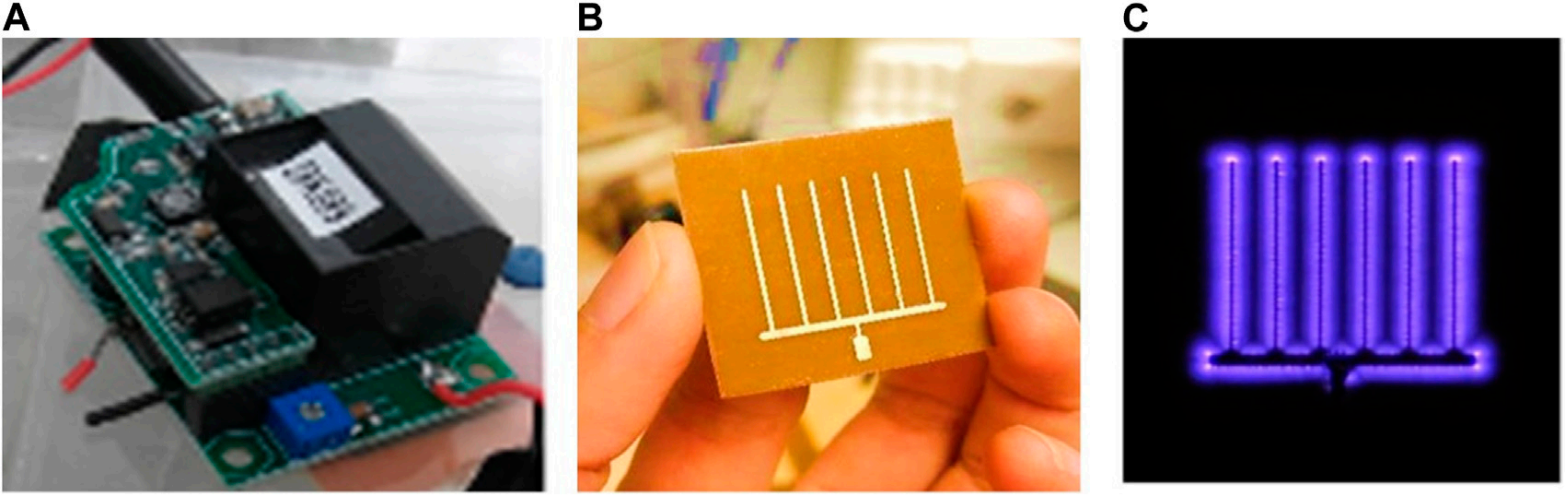
CPPR functional components **(A)** CPPR inverter circuit, **(B)** CPPR reactor panel and **(C)** Surface DBD on a powered CPPR.

The CPPR, also known as the Active Plasma Module, is a compact (48 cubic centimeters), lightweight (∼55 g), energy-efficient, and scalable device for generating surface Dielectric Barrier Discharge (DBD) (Choudhury et al., 2018). It uses the surrounding atmospheric air as the input gas and does not require additional air supply. The CPPR has 2 components: a) a reactor panel consisting of electrodes separated by a dielectric medium and b) a compact inverter circuit which converts low DC input voltage to high AC output voltage required for creating surface DBD on the reactor panel surface (Figure 1). Please refer to the introduction section for explanation of DBD and its role in ozone generation. The electrodes in the reactor panel were made of 35 μm-thick copper separated by a 0.76 mm-thick dielectric material: hydrocarbon/ceramic [RO4350B (Rogers Corporation, 2018)] composite with a dielectric constant of 3.48. The CPPRs in this contain a comb-shaped reactor panel which helps in distribution of the SDBD generated ozone by affecting the flow of the surrounding fluid (Choudhury et al., 2022). Although there can be other reactor panel designs for optimal distribution of ozone, they were out of scope for the current study. The CPPR is run by a 25 V DC power-supply (EMITEVER 24 V DC Power Supply; model: LX240125) with an average power consumption of 2.2 ± 0.37 W. Details of the power measurements can be found in a previously published study (Choudhury et al., 2018). The power supplies were encased in electrically insulating cases for added safety. 12 CPPR units were built to test their decontamination efficacies.

### 2.2 Experimental test chambers

**FIGURE 2:**
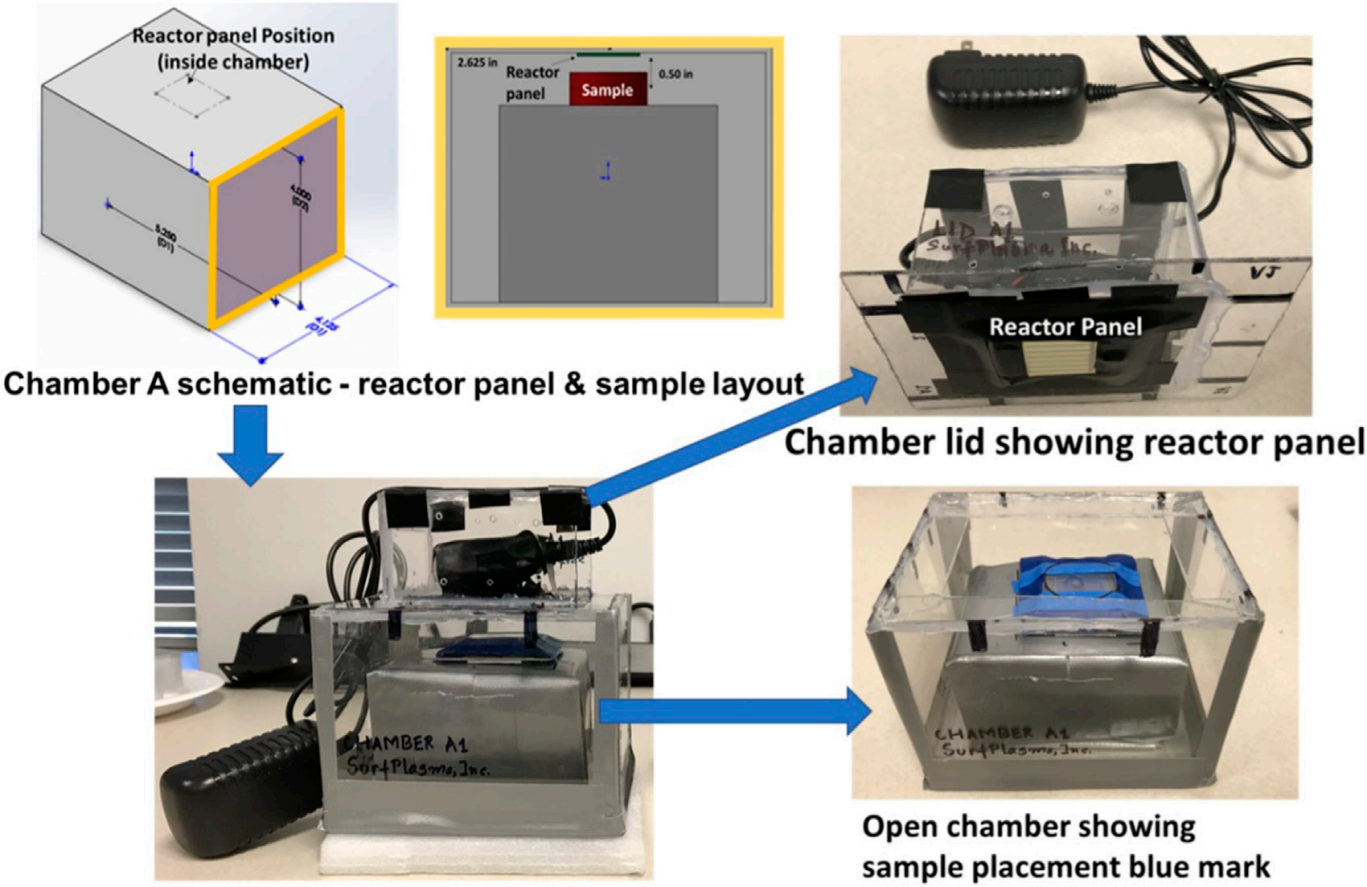
Chamber A schematics and photos showing reactor panel and contaminated sample placements. Chamber A is marked as Chamber A1 in the photo.

**FIGURE 3:**
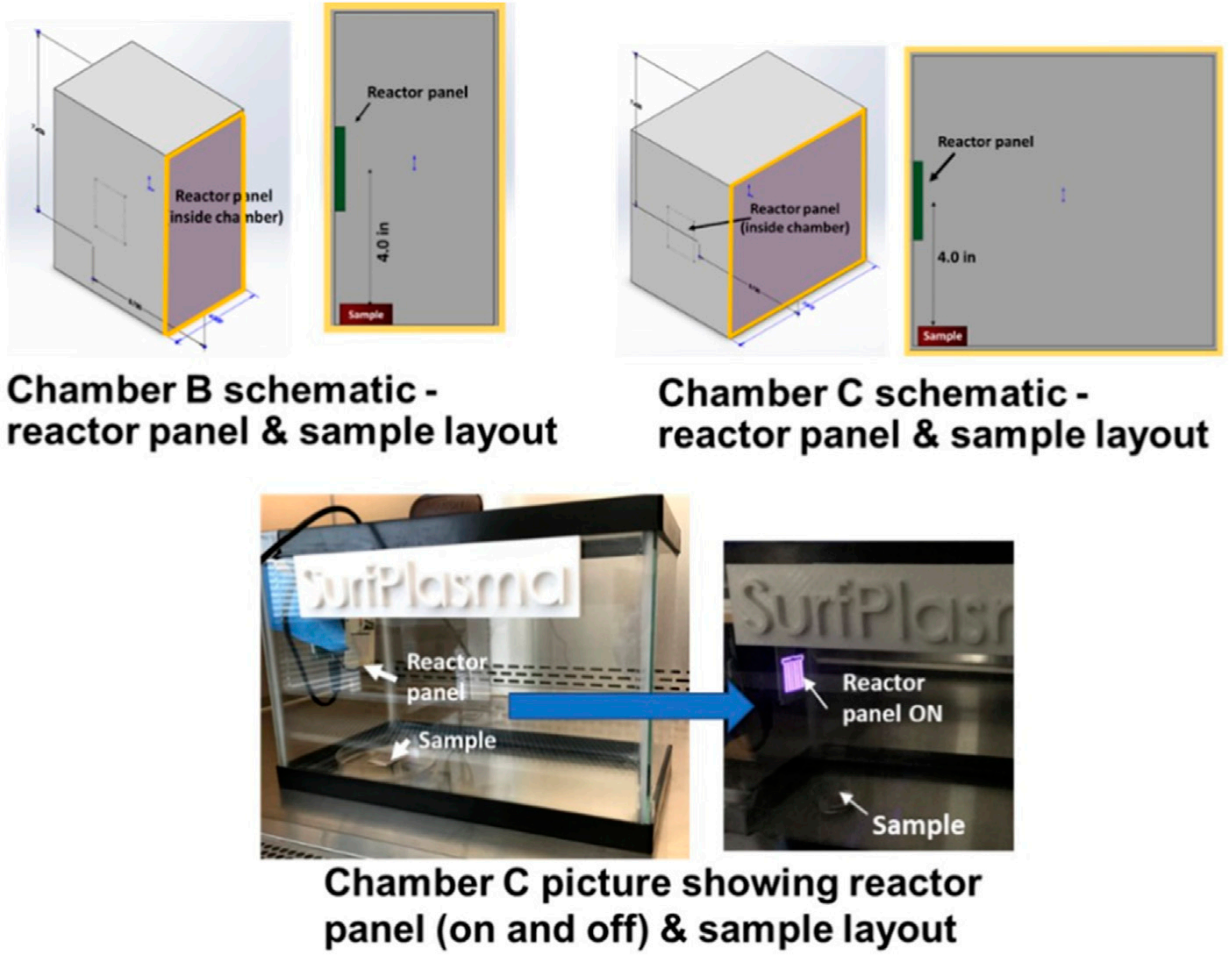
Chamber B and C schematics and photos showing reactor panel and contaminated sample placements.

Three experimental chambers were prepared to test decontamination achieved by one CPPR in three operating volumes - Chamber **A**: 0.0014 cu. m, Chamber **B**: 0.0028 cu. m. and Chamber **C**: 0.0056 cu. m. Figures 2, 3 show the schematics of the three chambers and the relative placement of the CPPR reactor panels and samples contaminated with the virus to be tested. The test chambers were manufactured at SurfPlasma, Inc. (nsf.gov). Experiments were performed in the three aforementioned test chambers with SARS CoV-2 and HCoV-OC43 on representative porous and nonporous materials. Iterative experiments on the surrogate virus was first performed to find the minimum exposure times required for inactivation by the CPPR in the three volumes. These exposure times were used to determine the initial exposure times for inactivation experiments on SARS CoV-2, followed by increments in exposure times till complete inactivation was achieved. This iterative approach of first testing the surrogate virus to determine the initial exposure times for inactivation experiments on SARS CoV-2 was important to reduce time in the BLS3 lab and save resources available for the project.

### 2.3 Cell cultures

Vero E6 cells (African green monkey kidney cells) were obtained from the American Type Culture collection (ATCC, Manassas, VA; catalog no. ATCC CRL-1586). The cells were propagated as monolayers in cell culture medium comprised of aDMEM (advanced Dulbecco’s modified essential medium, Invitrogen, Carlsbad, CA, United States) supplemented with 10% low-antibody, heat-inactivated, gamma-irradiated fetal bovine serum (FBS, Hyclone, GE Healthcare Life Sciences, Pittsburgh, PA, United States), L-alanine, L-glutamine dipeptide supplement (GlutaMAX), and 50 μg/mL penicillin, 50 μg/mL streptomycin, 100 μg/mL neomycin (PSN antibiotics, Invitrogen) with incubation at 37°C in 5% CO_2_.

### 2.4 Viruses

SARS-CoV-2 strain UF-1 and HCoV-OC43 strain JAL-1 were used for this project. The formal designation of SARS-CoV-2 strain UF-1 is SARS-CoV-2/human/USA/UF-1/2020, and its genomic sequence is available in GenBank (accession number MT295464.1) and at GISAID (accession no. EPI_ISL_424350) (Lednicky et al., 2020a). The SARS-CoV-2 used for this work was a second passage from the original isolate. This is a SARS-CoV-2 clade S (GISAID nomenclature) strain, which corresponds to Pango SARS-CoV-2 genetic Lineage A.1. The genome of SARS-CoV-2 UF-1 has a high nucleotide identity (29886/29892) with that of the original isolate (reference strain Wuhan Hu-1, GenBank accession number NC_045512.2). SARS-CoV-2 UF-1 was isolated in Vero E6 cells by Global Pathogen Discovery Lab (UF Dept. of Environmental and Global Health) from a COVID-19 patient at UF Health Shands Hospital, which is the UF teaching hospital in Gainesville, Florida. The complete genome was attained using next-generation sequencing in an Illumina MiSeq platform. No contaminating microorganism was identified through the sequencing process. The HCoV-OC43 strain used for this work was isolated from a human with rhinitis by the Global Pathogen Discovery Lab (UF Dept. of Environmental and Global Health), and has been fully sequenced using next-generation sequencing (Lednicky et al., 2020b). It was chosen for this work because it is readily propagated in Vero E6 cells, and is a BSL2-level pathogen.

### 2.5 Propagation and quantification of SARS-CoV-2 and HCoV-OC43

Both viruses were propagated in Vero E6 cells in cell culture medium comprised of a DMEM supplemented with antibiotics, 10% FBS, and GlutaMax at 37°C in 5% CO_2_. All laboratory work with SARS-CoV-2 was performed in a biosafety level 3 (BSL3) laboratory at the University of Florida (UF) Emerging Pathogens Institute (EPI). Virology procedures were performed in a Class II Type A2 Biosafety Cabinet (BSC). Analysts performing the work were expert at working with SARS-CoV-2, wore full head-covering powered air-purifying respirators, were garbed with disposable chemically impermeable Tyvek laboratory coats and other standard personal protective equipment (PPE), and the work was performed using BSL3 work practices (Lednicky et al., 2020a; Lednicky et al., 2020b). Laboratory work with HCoV-OC43 was performed in a BSL2 laboratory at the UF EPI. Virology procedures were performed in a Class II Type A2 BSC by trained analysts wearing personal protective equipment that included a Tyvek lab coat and disposable nitrile gloves.

### 2.6 Test materials

Representative porous (KN95 mask material) and nonporous materials (aluminum metal and polycarbonate plastic) were selected for decontamination tests—see Table 2 (xometry; chemicalbook; armbrustusa). Coupons were prepared by cutting the materials into 4 cm × 4 cm squares.

**TABLE 2:** Materials chosen for decontamination tests (Lednicky et al., 2020a; Lednicky et al., 2020b; xometry).

Materials	Material type and composition	Porosity
Aluminum 6060	Metal [(97.9%–99.3%) aluminum, (0.35%–0.5%) magnesium, (0.3%–0.6%) silicon, (0.1%–0.3%) iron, (0.10%) manganese, (0.05% max) chromium, (0.1% max) copper, (0.1% max) titanium, (0.15% max) zinc, and (0.15% max) residuals)]	Non-porous
Polycarbonate	Plastic (C_16_H_18_O_5_)	Non-porous
KN95 mask material	Layers of synthetic thermoplastic carbon polymers [5-Ply Protective Layers (2 Polypropylene spunbond layers, 3 meltblown non-woven electrostatic-charged layers]	Porous

### 2.7 Preparation of coupons for virus deposition

Work was performed in a Class II Type A2 BSC. After a precleaning step accomplished by wiping their surfaces with lint-less tissue paper, the coupons were sprayed with sterile deionized H_2_O, wiped dry with sterile tissue paper, then sprayed with sterile molecular-grade water, and wiped dry. They were then sprayed with 70% molecular grade ethanol to decontaminate their surfaces and left to air-dry, then placed into open sterile plastic petri dishes. The coupons in the petri dishes were then irradiated on each side for 5 min using the sterilizing UV light within the BSC for a more thorough surface decontamination step (Rudhart et al., 2022; Rutala et al., 2023). Post UV-irradiation, lids were placed on the petri dishes, which were then labelled and stored for experimental runs.

### 2.8 Deposition of viable virus onto coupons

**FIGURE 4:**
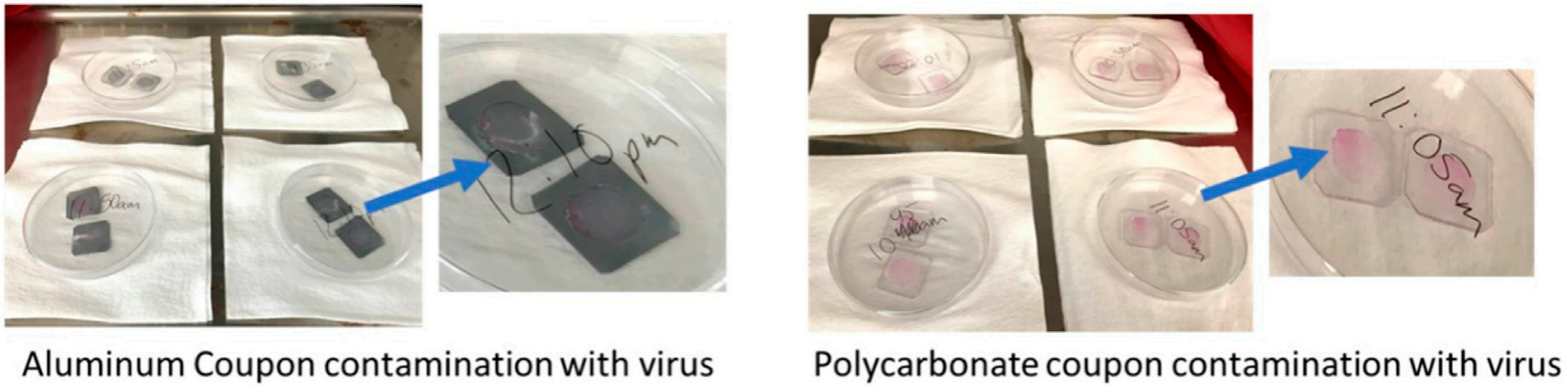
Deposition of SARS-CoV-2 and HCoV-OC43 onto coupon surfaces.

Work was performed in Class II Type A2 BSCs, in a BSL2 or BSL3 laboratory, as applicable. Coupons were aseptically placed atop a strip of sterile filter paper, after which 100 µL of a calibrated virus suspension containing 10^5^ plaque forming units (PFU) per ml was deposited and spread across the upper surface using a pipet tip, followed by covering the Petrie dish with its lid (Figure 4). The wet coupons were subsequently left to dry for 24 h in the BSC.

### 2.9 CPPR exposure experiments

Four coupons of each material, onto which 10^4^ PFU of virus had been deposited then dried onto the upper surface, were used per exposure experiment. For each trial, one of the coupons was positioned in the test chamber at a site demarcated by placement markers, then exposed to ozone for a specific exposure time. The remaining three coupons were placed outside the chamber for the same exposure times as controls. After the exposure periods, all 4 coupons were post-processed to determine virus survival counts expressed as PFUs/coupon. The following equation was used to calculate PFUs/coupon:

$$\text{PFUs}/\text{coupon}=\text{PFUs}/\text{ml }*\;V1=Dx\;*10^{x}*V1$$

where V1 = volume of cell culture media (mL) used to immerse coupons during post-processing, and Dx = PFUs counted in *x* ^th^ dilution plate. The reduction in virus quantity, expressed as PFUs obtained per coupon for each experiment, was determined from the difference in PFUs/coupon of the exposed and control (un-exposed) coupons. For statistical confidence, at least three repeats were performed for each data point. Virus inactivation is expressed as logs of reduction in PFUs/coupon.

### 2.10 Post-processing of coupons and determination of virus survival

Porous (KN95 mask material) and non-porous (aluminum and polycarbonate) coupons were post-processed differently to elute virus from the coupons. Porous material coupons were placed in a 50 mL conical tube with 15 mL of reduced serum DMEM, the tubes vortexed at mid-setting for 30 s, then left idle for 30 min at room temperature. This was followed by briefly centrifuging and transferring all the liquid into Amicon Ultra-15 centrifugal filter units with Ultracel-100 membranes with a molecular mass cutoff of 100 kDa (Millipore, Bedford, MA) which was centrifuged for 15 min at 4,000 × g. The liquid remining in the upper chamber of the concentrator tube was triturated against the filter to assist in dislodging virus particles stuck to the filter, and the liquid aseptically transferred into a sterile 1.5 mL cryotube, and recovered liquid volume adjusted to 1 mL by addition of DMEM with 3% FBS. For non-porous material coupons, 100 µL of cell culture medium was added onto the dried material on the coupons and the rewetted coupons replaced in the petri dishes for 30 min to help rehydrate the contents. Once the 30-min rehydration period had finished, the rewetted material was scraped into 900 µL of cell growth medium. Serial 10-fold dilutions of the recovered material were made, and aliquots inoculated onto Vero E6 cells for quantification of viable virus counts by plaque assays. Plaque assays were performed using 6-well tissue culture plates as per the method described in Ragan et al. (2020).

### 2.11 Ozone, temperature, and relative humidity measurements

The 2B Technologies Model 106–6 Ozone Monitor, which works based on UV light absorption at 254 nm, was used for the ozone measurements (twobtech). The monitor gives ozone measurements in units of ppm by volume. 1 ppm by volume equals 1 part per million by volume of ozone in a gaseous mixture as per EPA rule (EPA). 1 ppm by volume ozone equals to a concentration of 2.14 mg of ozone per cubic meter of air (Choudhury et al., 2018). Ozone concentrations reported in the results section are in ppm (by volume) in air and has been referred to as ppm for the rest of the paper. The accuracy of the monitor is 0.01 ppm or 2% of the reading. Ozone measurements were performed at the center of the decontamination box for the minimum required exposure times for inactivation (4–5 log reduction) determined through inactivation efficacy tests. Temperature and humidity were monitored with a Govee Hygrometer Thermometer (govee).

## 3 Results and discussion

### 3.1 Minimum exposure times

**FIGURE 5:**
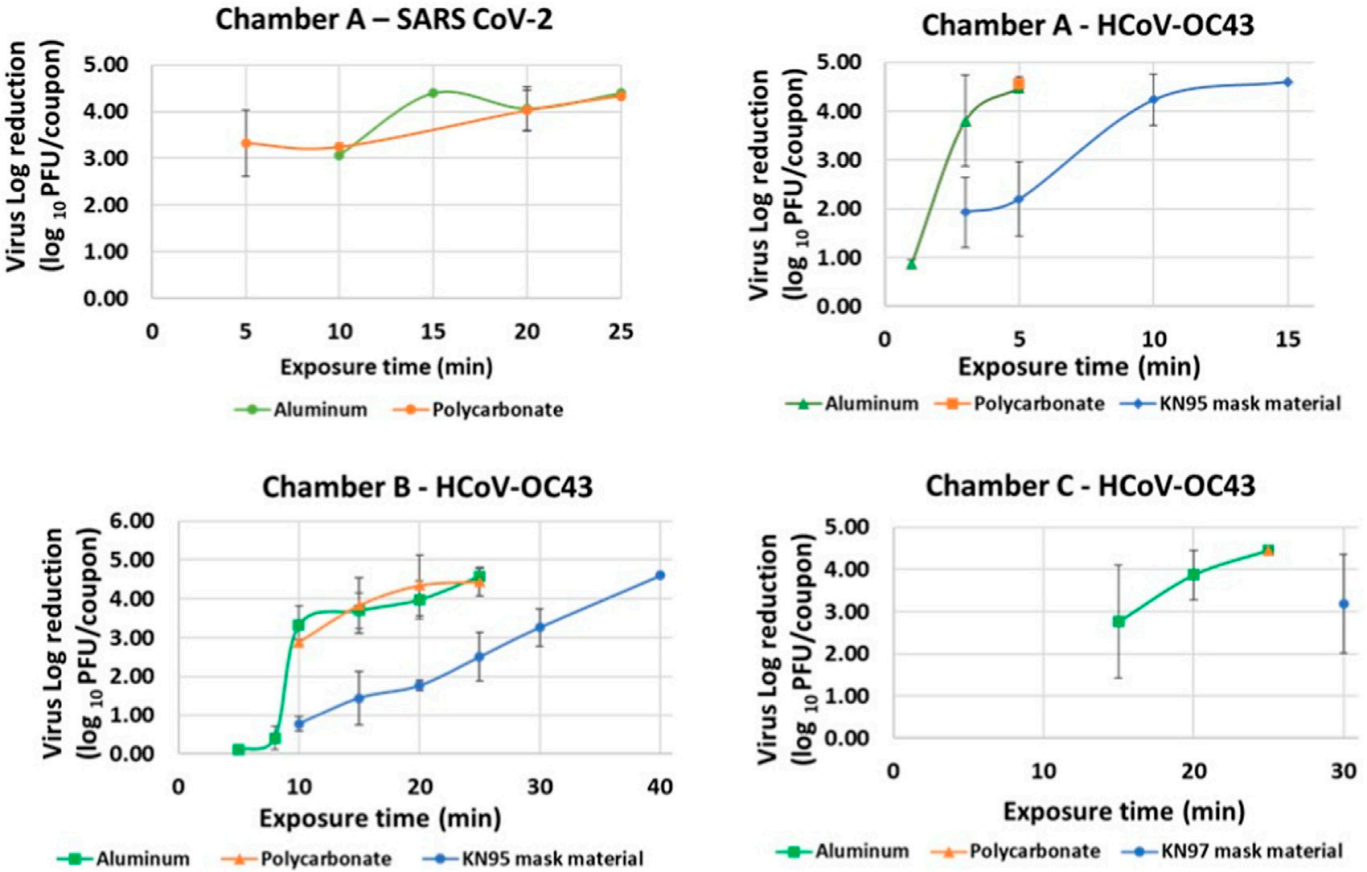
Virus inactivation achieved in iterative decontamination tests performed on contaminated Aluminum, Polycarbonate and KN95 mask materials in chambers A, B and C.

Iterative tests with varying exposure times were performed for each material type in Chambers A, B and C to determine minimum exposure times and establish complete killing. Killing of virus quantified by reduction in logs of PFUs/coupon calculated as the difference in PFUs/coupon in exposed and un-exposed coupons was plotted against time as shown in Figure 5. Tests were performed by increasing exposure times till complete killing was achieved. Complete killing refers to killing achieved when 0 PFUs/coupon were determined for exposed coupons. Table 3 shows the determined minimum exposure times obtained from the iterative tests. A dose-dependent virucidal effect was evident in an experiment performed with the two strains, three materials and three chambers. Chamber A data for SARS CoV 2 and HCoV-OC43 inactivation was used to relate CPPR killing of SARS CoV 2 and HCoV-OC43 for the material types and the operating volumes. Please note that SARS CoV 2 was only tested on the non-porous materials in chamber A due to limitation in resources and is planned for the next phase of this study. Similar exposure times were required for the inactivation of the viruses on the non-porous material types, in contrast to relatively longer exposure times was required to decontaminate porous material type.

**TABLE 3:** Minimum exposure times for complete killing using one CPPR in three test chambers.

	Exposure time for complete killing (4–5 log reduction)			
Material/Chamber	Chamber A		Chamber B	Chamber C
	SARS CoV-2	HCoV-OC43	HCoV-OC43	HCoV-OC43
Aluminum	15 min	5 min	25 min	25 min
Polycarbonate	15 min	5 min	25 min	25 min
KN95 mask material	-	15 min	40 min	50 min

### 3.2 Inactivation data (complete killing resulting in 4–5 log reduction)

**FIGURE 6:**
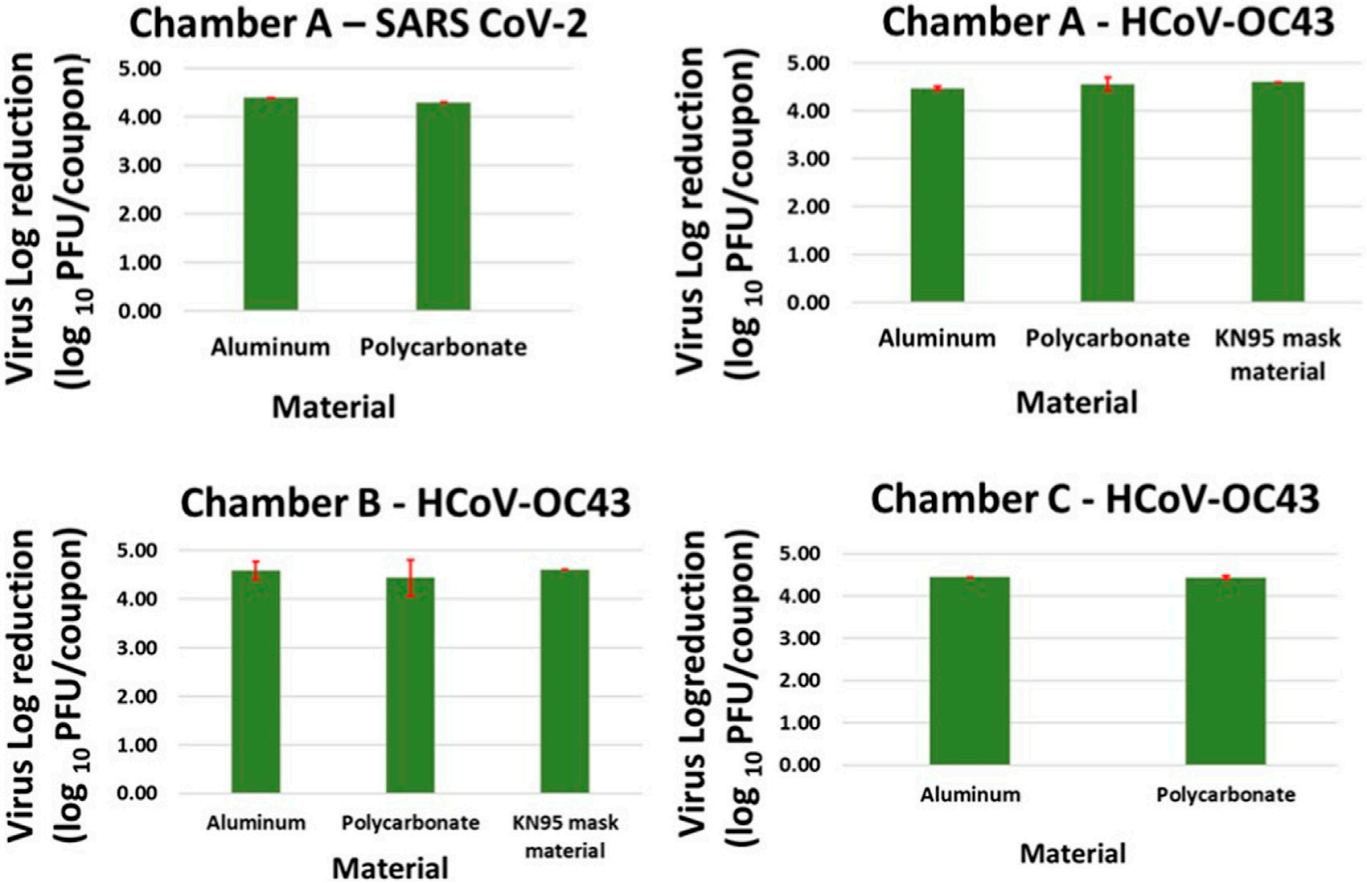
Complete killing (4–5 log reduction) of virus achieved on Aluminum, Polycarbonate and KN95 mask materials in chamber A within 15 min, and in chamber B and C within 40 min. The error bars are based on standard deviation calculated from 3 repeats for each data point.

Complete inactivation (4–5 log reduction) was achieved for SARS CoV-2 contaminated Aluminum and Polycarbonate coupons in Chamber A within 20 min. Complete killing (4–5 log reduction) was achieved on HCoV-OC43 contaminated Aluminum and Polycarbonate coupons in Chambers A, B and C within 5, 25, and 25 min, respectively. Complete inactivation of HCoV-OC43 was achieved on KN95 mask materials in chambers A and B within 15 and 40 min, respectively. Results are shown in Figure 6.

### 3.3 Ozone requirements

**FIGURE 7:**
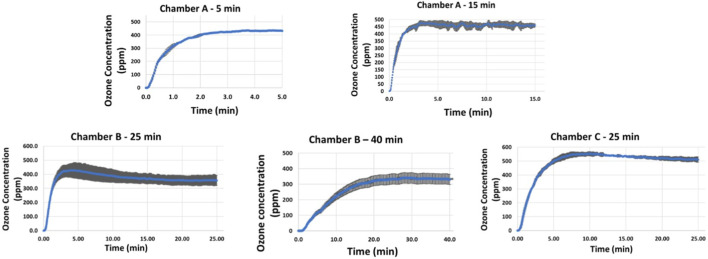
Ozone concentrations corresponding to exposure times required for complete killing (4–5 log reduction) achieved on Aluminum, Polycarbonate and KN95 mask materials in chamber A (5–15 min), chamber B (25–40 min), and chamber C (40 min).

Based on the exposure times that resulted in 4–5 reductions in SARS CoV 2 and HCoV-OC43 concentrations on coupons (PFUs/coupon), ozone data was collected for the following exposure times in the 3 chambers: a) Chamber A: 5 and 15 min, b) Chamber B: 25 and 40 min and c) Chamber C: 25 min. The results are shown in Figure 7. Two to three repeats were performed for each exposure time to gain statistical confidence and standard deviation observed in those repeats were used to represent error bars in the graph. Variations in ozone concentrations measured at the center of the 3 chambers at different times can be attributed to the difference in test chamber volumes, variations in atmospheric air conditions (Nazaroff and Weschler, 2022) and limitations associated with experimental ozone measurements due to unstable nature of ozone that result in decomposition of ozone to oxygen (Choudhury et al., 2022; Lin et al., 2023). Minimum ozone concentrations required to obtain complete inactivation (4–5 log reduction) of SARS-CoV-2/surrogate was found to be 300–550 ppm based on material type and chamber volume.

### 3.4 Energy requirements

The CPPR energy requirement for decontamination of SARS CoV-2 and HCoV-OC43 in the 3 test chambers was calculated as the product of power requirement of a single CPPR—2.2 ± 0.37 W (Choudhury et al., 2018), and the exposure times reported in the previous sections. For an exposure time of 5 min, the energy requirement was calculated as 2.2 W × 5 min which would equal to 660 J or 0.6 kJ. A range of CPPR energy requirements is given for each chamber based on the minimum and maximum exposure times required for inactivating the virus in 3 sample test materials. The CPPR energy requirements are given in Table 4.

**TABLE 4:** CPPR energy requirements for complete killing using one CPPR in three test chambers.

Test chamber	Min exposure time (min)	Max exposure time (min)	Range of energy requirements (kJ)
A	5	15	0.6–1.9
B	25	40	3.3–5.2
C	25	50	3.3–6.6

### 3.5 Effect of material type and chamber volume on exposure times required for inactivation

Complete killing (4–5 log reduction) of HCoV-OC43 on inoculated K95 mask material samples required higher exposure times than on inoculated Aluminum and Polycarbonate samples in chambers A (15 min vs. 5 min) and B (25 min vs. 40 min). This can be attributed to the additional time required for ozone to penetrate through layers of synthetic thermoplastic carbon polymers and inactivate the virus which might have percolated through the outermost layer. Further, when equal amount of calibrated virus suspension with 10^5^ PFUs/mL was deposited and spread across the K95 mask material sample, the inoculation volume was visually observed to be partially absorbed by the mask material sample, whereas no such observation was made for aluminum and polycarbonate samples. This can lead to longer exposure times required for disinfecting the K95 mask material sample as it makes it more difficult for the ozone to reach the virus across the porous mask material sample in comparison to the non-porous material samples. Future research with extensive testing of various material samples is needed to support conjectures discussed above to explain the difference seen in required CPPR exposure times for disinfecting porous and non-porous materials. The scope of this feasibility study is limited to establishing the efficacy of the CPPR in disinfecting sample porous and non-porous materials inoculated with SARS CoV-2 and its surrogate HCoV-OC43.

In terms of chamber volume, HCoV-OC43 inactivation (4–5 log reduction) data obtained for Chambers B and C for non-porous materials (Aluminum, and Polycarbonate) shows that an exposure time of 25 min was required for decontamination in both chamber volumes. In contrast, HCoV-OC43 inactivation (4–5 log reduction) for both non-porous materials was achieved within 5 min in Chamber A. Note that the distance between reactor panel and inoculated material sample was same (4 inches) in all Chamber B and C tests, whereas this value was lower for Chamber A (0.5 inch). These results suggest that chamber volume is not a significant factor for determining required exposure times. However, distance between reactor panel and inoculated sample is observed to affect required exposure time inversely.

### 3.6 Effect of relative humidity and temperature

**FIGURE 8:**
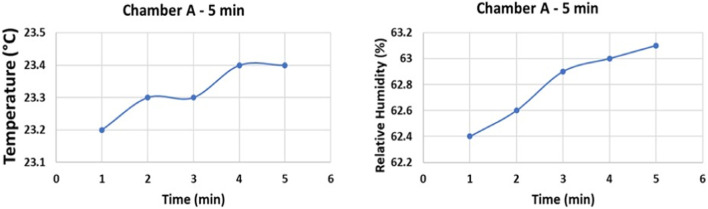
Temperature and relative humidity data of Chamber A experiments on Aluminum and Polycarbonate.

In operating temperatures of 21°C–30°C, variation of relative humidity from 45% to 90% in repeated experiments in all 3 chambers, with fixed exposure times, did not show significant effect on disinfection of SARS-CoV-2 and surrogate contaminated surfaces using the CPPR. Figure 8 shows the variation in temperature and relative humidity for 5 min exposure experiments performed in Chamber A.

### 3.7 Preliminary material compatibility

Visual inspection of color and texture of CPPR exposed material coupons did not show significant material damage. Material compatibility studies with SEM (Scanning Electron Microscopy) imaging for CPPR decontamination has been reported in a previously published paper (Choudhury et al., 2023). Further, there is literature available on material compatibility of ozone for decontamination purposes (Epelle et al., 2023a). Researchers also suggest that ozone exposure for decontamination of N95 respirators does not lead to significant change in filtration efficacy, fit and change in strap integrity (Manning et al., 2020). Despite the literature available on compatibility of materials for CPPR and ozone decontamination, there is still a need of future research to establish a baseline for decontamination of various microbes under different operating conditions based on the application.

## 4 Conclusion

This paper demonstrates the efficacy of an alternate decontamination technology - the Compact Portable Plasma Reactor (CPPR) for inactivation (4–5 log reduction) of SARS CoV-2 and its surrogate—Human coronavirus OC43 (HCoV-OC43) on representative porous (KN95 mask material) and nonporous materials (Aluminum metal and Polycarbonate plastic) in 3 operating volumes. The CPPR exposure time required for inactivation of SARS CoV-2 in an operating volume of 0.05 cu. ft. (Chamber A) was found to be 15 min while that required for inactivation of its surrogate HCoV-OC43 was found to be 5 min under the same operating conditions. This indicates that SARS CoV-2 inactivation has higher exposure time and ozone concentration requirements compared to its human surrogate HCoV-OC43. Additionally, the CPPR exposure time required for inactivation of HCoV-OC43 on both non-porous materials in operating volumes of 0.1 cu. ft and 0.2 cu. ft (Chambers B and C) was found to be 25 min. In contrast, inactivation of HCoV-OC43 on the selected porous material in the operating volumes of 0.1 cu. ft and 0.2 cu. ft was found to be 40 min and 50 min, respectively. This suggests that the CPPR exposure times and resulting ozone dosage required for decontaminating porous materials is higher than the requirements of non-porous materials; and can be dependent on the operating volume. Minimum ozone concentrations required to obtain complete inactivation (4–5 log reduction) of SARS-CoV-2/surrogate was found to range between 300 and 550 ppm based on material type and chamber volume. Energy required to power up the CPPR for complete inactivation of the viruses ranged from 0.6 to 6.6 kJ depending on the material type, and operating volume. Furthermore, inactivation data of HCoV-OC43 in the 3 operating volumes for non-porous materials implies that distance between reactor panel and inoculated sample has a greater impact on required CPPR exposure times than the operating volumes. Future research involves performing decontamination tests with other BSL-2 and BSL-3 pathogens of interest, extensive scaling studies, ozone dosage testing and material compatibility tests for developing the CPPR as a sterilizer.

In conclusion, the results of this feasibility study show the potential of the CPPR as a powerful, scalable, decontamination technology for reducing the spread of infection diseases like COVID-19 and future pandemics. Additionally, previous literature on pathogen decontamination using the CPPR specifically (Choudhury et al., 2018; Roy et al., 2021; Choudhury et al., 2023), and DBD based ozone in general (Kogelschatz, 2003; Laroussi and Leipold, 2004; Park et al., 2006; Mastanaiah et al., 2013; Scholtz et al., 2015; Mandal et al., 2018; Gradini et al., 2019; Office of Planetary Protection, 2019; Bayarri et al., 2021; Feizollahi et al., 2021; Bhartiya et al., 2022; Choudhury et al., 2022; Ashokkumar et al., 2023; Epelle et al., 2023b; Kaushik et al., 2023), supports the feasibility of CPPR technology against various pathogens bacterial and fungal species. Thus, broader implications for public health lie in the potential of the CPPR to address the societal need of a non-thermal (low processing temperatures), convenient, portable, economical, safe and efficient solution for disinfecting PPE, surgical tools, medical devices, food, beverages, etc. contaminated with harmful pathogens. The CPPR technology has the potential to be an effective decontamination solution to combat the spread of infectious diseases during future outbreaks like the COVID-19, in austere military medical environments, and in countries with limited resources. The major impact is expected in crowded facilities and community settings with limited resources where rapid disinfection of objects is required.

## Data Availability

The original contributions presented in the study are publicly available. This data can be found here: https://www.ncbi.nlm.nih.gov/nuccore/MT295464.1.
